# HT-SIP: a semi-automated stable isotope probing pipeline identifies cross-kingdom interactions in the hyphosphere of arbuscular mycorrhizal fungi

**DOI:** 10.1186/s40168-022-01391-z

**Published:** 2022-11-25

**Authors:** Erin E. Nuccio, Steven J. Blazewicz, Marissa Lafler, Ashley N. Campbell, Anne Kakouridis, Jeffrey A. Kimbrel, Jessica Wollard, Dariia Vyshenska, Robert Riley, Andy Tomatsu, Rachel Hestrin, Rex R. Malmstrom, Mary Firestone, Jennifer Pett-Ridge

**Affiliations:** 1 Physical and Life Sciences Directorate, Lawrence Livermore National Laboratory, Livermore, CA USA; 2 Biosciences Division, Lawrence Berkeley National Laboratory, Berkeley, CA USA; 3 Department of Environmental Science Policy and Management, University of California, Berkeley, CA USA; 4 DOE Joint Genome Institute, Berkeley, CA USA; 5 Stockbridge School of Agriculture, University of Massachusetts, Amherst, MA USA; 6 Life & Environmental Sciences Department, University of California Merced, Merced, CA USA

**Keywords:** Stable-isotope probing, Metagenomics, Microbial community, Arbuscular mycorrhizal fungi, SIP, AMF, Soil, Bacteria, Archaea, Ammonia oxidation

## Abstract

**Background:**

Linking the identity of wild microbes with their ecophysiological traits and environmental functions is a key ambition for microbial ecologists. Of many techniques that strive for this goal, Stable-isotope probing—SIP—remains among the most comprehensive for studying whole microbial communities in situ. In DNA-SIP, actively growing microorganisms that take up an isotopically heavy substrate build heavier DNA, which can be partitioned by density into multiple fractions and sequenced. However, SIP is relatively low throughput and requires significant hands-on labor. We designed and tested a semi-automated, high-throughput SIP (HT-SIP) pipeline to support well-replicated, temporally resolved amplicon and metagenomics experiments. We applied this pipeline to a soil microhabitat with significant ecological importance—the hyphosphere zone surrounding arbuscular mycorrhizal fungal (AMF) hyphae. AMF form symbiotic relationships with most plant species and play key roles in terrestrial nutrient and carbon cycling.

**Results:**

Our HT-SIP pipeline for fractionation, cleanup, and nucleic acid quantification of density gradients requires one-sixth of the hands-on labor compared to manual SIP and allows 16 samples to be processed simultaneously. Automated density fractionation increased the reproducibility of SIP gradients compared to manual fractionation, and we show adding a non-ionic detergent to the gradient buffer improved SIP DNA recovery. We applied HT-SIP to ^13^C-AMF hyphosphere DNA from a ^13^CO_2_ plant labeling study and created metagenome-assembled genomes (MAGs) using high-resolution SIP metagenomics (14 metagenomes per gradient). SIP confirmed the AMF *Rhizophagus intraradices* and associated MAGs were highly enriched (10–33 atom% ^13^C), even though the soils’ overall enrichment was low (1.8 atom% ^13^C). We assembled 212 ^13^C-hyphosphere MAGs; the hyphosphere taxa that assimilated the most AMF-derived ^13^C were from the phyla Myxococcota, Fibrobacterota, Verrucomicrobiota, and the ammonia-oxidizing archaeon genus *Nitrososphaera*.

**Conclusions:**

Our semi-automated HT-SIP approach decreases operator time and improves reproducibility by targeting the most labor-intensive steps of SIP—fraction collection and cleanup. We illustrate this approach in a unique and understudied soil microhabitat—generating MAGs of actively growing microbes living in the AMF hyphosphere (without plant roots). The MAGs’ phylogenetic composition and gene content suggest predation, decomposition, and ammonia oxidation may be key processes in hyphosphere nutrient cycling.

Video Abstract

**Supplementary Information:**

The online version contains supplementary material available at 10.1186/s40168-022-01391-z.

## Background

Stable-isotope probing “SIP” approaches—where active microbes are identified via incorporation of stable isotopes into their biomass—are among the most powerful methods in microbial ecology since they can identify active microbes and their ecophysiological traits (substrate use, cellular biochemistry, metabolism, growth, mortality) in complex communities under native, uncultured conditions [1–7]. Broadly speaking, SIP refers to any technique where microorganisms that have consumed a substrate enriched in a rare stable isotope (e.g., ^13^C, ^15^N, ^18^O) are identified based on the resulting isotopic enrichment of their nucleic acids, proteins, lipids, and metabolites (reviewed in [5, 8]). However, DNA-SIP—where isotopically enriched DNA is separated from unenriched nucleic acids via isopycnic separation in cesium chloride—is one of the most commonly used SIP approaches, typically in conjunction with 16S rRNA gene or shotgun metagenome analysis. A cornerstone of many seminal studies of microbial biogeochemical cycling, DNA-SIP has been used to identify communities that consume plant root exudates and structural compounds [9–11], degrade pollutants and C_1_ compounds (e.g., methanol) [12, 13], oxidize ammonia [14], and fix N_2_ [15, 16], and it also has been used to characterize population growth, survival, and mortality in mixed communities [17, 18].

Quantitative stable-isotope probing (qSIP) and techniques such as “high-resolution” SIP (HR-SIP) [19] are expansions of the original SIP concept that combine density gradient ultracentrifugation with mathematical models designed to improve the quantification of isotope enrichment [6, 20]. qSIP enables taxon-specific estimates of isotope enrichment and substrate consumption (using ^13^C and ^15^N) [21, 22], as well as cell growth and mortality rates of individual taxa (using ^18^O-enriched water) [23–25]. Recent qSIP studies have used the method to illustrate how wild microbial communities are shaped by evolutionary history [26, 27], soil temperature and warming [28, 29], amendments of water and nutrients [18, 30], and trophic relationships among bacterial predators and their prey [31].

While the majority of SIP studies have focused on 16S rRNA gene profiles, targeting active populations with shotgun sequencing (metagenomes and metatranscriptomes) provides greater opportunity to infer genomic potential and function [32–34], define microbial guilds [35], and provide insights into cross-kingdom interactions (including virus-host matching) [25, 36]. But SIP metagenomics is a daunting prospect for many research groups, in part because processing SIP density gradients is a relatively low-throughput process and requires significant hands-on labor. Historically, SIP studies have used few replicates due to the laborious nature of the technique. To address this short-coming, we have designed a high-throughput SIP (HT-SIP) pipeline (replicated at both LLNL and the JGI) for processing SIP density gradients, which automates fractionation, partially automates fraction cleanup, and automates the preparation of samples for nucleic acid quantification. Since establishing our pipeline, over the course of 1.5 years with one primary technician, we have run more than 1000 samples from a diverse array of sites, including samples from boreal, temperate grassland [33], agricultural [37], and tropical forest habitats.

Here, using our HT-SIP pipeline, we targeted an important yet challenging sample set—the “hyphosphere” microhabitat— the soil immediately surrounding arbuscular mycorrhizal fungal (AMF) hyphae. Arbuscular mycorrhizal fungi (members of the Glomeromycota and Mucoromycota) form obligate symbiotic associations with 72% of all land plants [38], and in exchange for plant carbon (C), supply their host with essential nutrients such as N and P [39, 40] and water [41]. Intriguingly, AMF are capable of stimulating decomposition of soil organic matter (SOM) and dead plant material [42–44], but do not have the enzymatic repertoire to decompose SOM themselves. As such, interactions with the soil microbiome are potentially critical [45], and previous research suggests that AMF modify their surrounding soil litter-decomposing microbial community in order to acquire N derived from SOM, and transport it to their host plant [40, 46]. However, these interactions occur at such a small spatial scale (hyphae are ca. 1.5–18 μm in diameter [47]) that they are extremely difficult to measure and monitor.

Using HT-SIP and SIP metagenomics, we tracked plant-fixed ^13^CO_2_ through AMF hyphae and into the ^13^C-hyphosphere microbiome. The wild annual grass, *Avena barbata*, was inoculated with the AMF *Rhizophagus intraradices* in sterile sand in two-compartment microcosms. The microcosms contained a separate hyphal compartment with live soil (non-sterile) that excluded roots but permitted fungal hyphae. In this soil, visible hyphae and associated soil particles were collected and extracted for ^13^C-hyphosphere SIP processing. We used our semi-automated pipeline to process samples from this microhabitat, which produced high-quality libraries and MAGs even though the hyphosphere soil had overall low isotopic enrichment (1.8 atom% ^13^C), and we used an unusually low starting DNA input for SIP separations (350 ng DNA). Our work demonstrates that automation not only saves operator time and improves reproducibility of SIP processing, but is also suitable for analysis of low DNA quantities and downstream amplicon and metagenomics analysis. The ^13^C-hyphosphere MAGs assembled in this study are a key advance for dissecting trophic interactions in the AMF hyphosphere.

## Methods

### Density gradient separations

HT-SIP validation experiments were conducted using 1–5 μg DNA for SIP density gradient separations (below, amounts and DNA sources are specified per experiment). To separate DNA based on isotopic enrichment, DNA was added to 150 μL 1xTE buffer mixed with 1.0 mL gradient buffer, and 4.6 mL cesium chloride (CsCl) stock (1.885 g mL^−1^) with a final density of 1.725–1.730 g mL^−1^. Samples were loaded into ultracentrifuge tubes (5.1 mL, Quick-Seal Round-Top Polypropylene Tube, Beckman Coulter), and density gradients were created as previously described [17, 48] by centrifuging for 108 h at 176,284 RCF_avg_ (equivalent to 176,284×*g*) at 20 °C in a Beckman Coulter Optima XE-90 ultracentrifuge using a VTi65.2 rotor.

### High-throughput SIP (HT-SIP) pipeline

To automate the labor-intensive steps of SIP—density gradient fractionation, cleanup, and quantification—we combined a series of robotic instruments. Following CsCl density gradient separation in an ultracentrifuge, we automated fractionation by connecting an Agilent Technologies 1260 Isocratic Pump and 1260 Fraction Collector to a Beckman Coulter Fraction Recovery System (see Supplemental Figure S1 for schematic and parts list). In this system, each gradient is separated into 22 fractions (~236 μL each). CsCl is displaced in the ultracentrifuge tube by pumping sterile water at 0.25 mL min^−1^ through a 25G needle inserted into the top of the ultracentrifuge tube, and the sample fraction exits via a side port needle inserted into the bottom of the tube. We maintain pressure between 1 and 1.8 bar; pressures above this indicate the system is clogged. The CsCl gradient medium is then dispensed into 96-well deep-well plates (2 ml square well plates with v-bottoms, Stellar Scientific) by the Agilent Fraction Collector. Four SIP tubes are fractionated into a single deep-well plate (88 wells) and the final row is left empty for PicoGreen quantification standards. At the beginning of the day and after every four gradients, we clean the fractionation tubing with water using a “wash spacer” to bypass the fraction recovery system (see Supplemental Figure S1). The density of each fraction is measured manually using a Reichart AR200 digital refractometer fitted with a prism covering to facilitate measurement from 5 μL, as previously described [49].

DNA in each density fraction is then purified (desalted) and concentrated using a Hamilton Microlab STAR liquid handling robot, which we have programmed to partially automate PEG precipitations using a previously published protocol [50], with modifications for 96-well plates. We configured our robot deck to process four plates; this allows a maximum of 16 SIP samples to be processed simultaneously (4 samples per plate). Following fractionation, the robot adds 2 volumes of 30% PEG 6000 (in 1.6 M NaCl) and 35 μl of 1:5 diluted Glycoblue (Invitrogen, Thermo Fisher) to each well. Plates are manually sealed and mixed thoroughly by vortexing and manual shaking, pulsed down briefly, and incubated at room temperature in the dark overnight. To precipitate the DNA, we spin the four plates at 4198 RCF for 5 h at 20°C in an Eppendorf 5920R centrifuge using a S-4xUniversal-Large rotor. The plates are then placed back in the Hamilton robot, which removes the PEG by pipetting and rinses the pellets using 950 μl 70% ethanol. Plates are manually sealed, gently mixed by vortexing, and centrifuged at 4198 RCF for 1.5 h at 20°C to stabilize the DNA pellets. The robot removes the ethanol, and the plates are manually placed upside down on a paper towel to drain remaining ethanol. The plates are then returned to the robot to dry for 15 min, whereafter the robot automatically resuspends the DNA pellets in 40 μL of 1× Tris-EDTA (pH 7.5); 10 mM Tris-HCl may be used for applications sensitive to EDTA. Plates are manually sealed and stored at −20°C.

Finally, the DNA concentration of each fraction is quantified with a PicoGreen fluorescence assay (Invitrogen, Thermo Fisher). Picogreen quantification plates are prepared in triplicate on a Hamilton Microlab STAR robot, where each plate contains a row for the standard curve. Samples are mixed with the PicoGreen reagent in a 96-well intermediate mixing plate, and then distributed into three 96-well PCR plates for fluorescence analysis. Plate fluorescence is measured in a CFX Connect Real-Time PCR Detection System (Bio-Rad), and the fluorescence values for the three technical replicate plates are averaged to determine DNA concentration.

### Validation of HT-SIP using manual SIP

To validate the automated steps of our HT-SIP pipeline, we compared fractionation and PEG precipitations using both manual and automated methods. Automated fractionation was performed as described above, and manual fractionation was conducted with a Beckman Coulter fraction recovery system as previously described [17]. Samples were fractionated into approximately 22 fractions, although the number of fractions recovered by manual SIP typically varies despite identical run conditions.

To compare automated versus manual PEG precipitations, 4 μg soil DNA (extracted from a sample collected at the Hopland Research and Extension Center in Hopland, CA 38° 59′ 35″ N, 123° 4′ 3″ W) was added per density gradient. Automated precipitations were performed as described above. For manual precipitations, PEG precipitations were conducted in microcentrifuge tubes as previously described [50] using published centrifuge speeds and times, which we note are faster than those used for our HT-SIP plate-based method.

### Increasing DNA-SIP recovery using non-ionic detergents

Absorption of DNA to polypropylene tubes can lead to substantial sample loss, especially for DNA in high ionic strength solutions, but this concern can be mitigated by adding non-ionic detergents [51]. Since the ultracentrifuge tubes used in DNA-SIP protocols are made of polypropylene and CsCl is a high ionic strength solution, we tested whether adding the non-ionic detergents Tween-20 and Triton-X to density gradient buffer improved DNA recovery. To identify the optimal concentration of detergent for DNA-SIP recovery, we tested how Tween-20 additions (range 0.0001–1%) and Triton-X additions (range 0.0001–0.1%) affected DNA recovery versus the standard density gradient formulation. One microgram of *E. coli* genomic DNA (Thermo Scientific) was added to density gradients and processed using the HT-SIP pipeline (*n*=3 gradients per condition). Excel was used to perform Student’s two-tailed *t* tests to compare the detergent additions with the no-addition control.

After identifying that adding 0.0001% Tween-20 had the highest percent DNA recovery (Supplemental Table S1), we used our HT-SIP pipeline to assess how adding 0.0001% Tween-20 to a larger set of soil DNA samples (101 SIP tubes total) affected DNA recovery. We added 4 μg of soil DNA (from Hopland, CA soil) to these gradients.

### Application of HT-SIP pipeline: hyphosphere ^13^CO_2_ labeling and harvest

AMF hyphosphere soil was labeled in ^13^CO_2_ plant growth chambers; details on the microcosm design and growth conditions are documented in [41]. Briefly, *Avena barbata* seedlings were planted in the “plant compartment” of two-compartment microcosms and grown for 10 weeks (see Supplemental Figure S2 for microcosm design). The plant compartment was separated from the “no-plant compartment” by a 3.2-mm air gap to prevent root exudates or dissolved organic C from traveling via mass flow between the compartments. Each compartment was 10 × 2.5 × 26.5 cm (W × D × H), and both sides of the air gap had nylon mesh that either allowed hyphae but excluded roots (18 μm mesh), or that excluded both hyphae and roots (0.45 μm mesh).

In the plant compartment, a sand mix (1:1 volumes of triple-autoclaved sand and clay, plus 78 mg of autoclaved bone meal [52]) was inoculated with 26 g of “whole” inoculum of *Rhizophagus intraradices* (accession number AZ243, International Culture Collection of (Vesicular) Arbuscular Mycorrhizal Fungi (INVAM), West Virginia University, Morgantown, WV); this whole inoculum is mono-mycorrhizal and initially propagated in sterile media, but was not bacteria-free at the time of harvest. For the sand mix, 48 h elapsed between each autoclave treatment (3 total) to destroy spores that may have germinated after the first autoclave treatment. The no-plant compartment contained a mixture of 1:1 volumes of live soil (from Hopland CA) and sand plus 78 mg of autoclaved bone meal.

The microcosms were incubated in temperature-controlled growth chambers at the UC Berkeley Environmental Plant Isotope Chamber (EPIC) facility, which has a multiplexed ^13^CO_2_ delivery system monitored by an infrared gas analyzer (IRGA) and a Picarro CO_2_ analyzer; the IRGA was used in this experiment. The labeling chamber was supplied with 99 atom% ^13^CO_2_ and maintained at approximately 400 ppm CO_2_. For this study, three microcosms with 18 μm mesh (with^ 13^C-AMF permitted in the no-plant compartment, termed ‘^13^C-AMF’) and three microcosms with 0.45 μm mesh (with ^13^C-AMF excluded from the no-plant compartment, termed “^13^C no-AMF control” and used for isotope ratio mass spectrometry (IRMS) analysis only) were continuously ^13^CO_2_-labeled for 6 weeks during weeks 5–10 of plant growth (see Supplemental Figure S2 for labeling conditions). Six additional microcosms remained in a natural abundance ^12^CO_2_ atmosphere for the full 10 weeks; of these, the three ^12^C microcosms with 18 μm mesh (^12^C AMF permitted in the no-plant compartment, termed ‘^12^C-AMF’) served as the ^12^C-AMF SIP controls, and three ^12^C microcosms with 0.45 μm mesh (^12^C AMF excluded from the no-plant compartment, termed “^12^C no-AMF control”) were used for IRMS analysis only. AMF-specific Sanger sequencing of the plant compartment (roots, sand) as well as the air gap indicated the planted compartment contained the initial mycorrhizal inoculum [41].

At the beginning of week 11, all microcosms were destructively sampled. Immediately after harvest, soil from the no-plant compartment was flash frozen in liquid nitrogen and stored at −80°C. Plant shoots, roots, and soil from the plant and no-plant compartments were placed in paper envelopes and then dried at 60°C for 72 h for ^13^C IRMS analysis. These samples were finely ground, weighed, and analyzed for total C and ^13^C abundance by dry combustion on a PDZ Europa ANCA-GSL elemental analyzer interfaced to a PDZ Europa 20-20 isotope ratio mass spectrometer (Sercon Ltd., Cheshire, UK). Stated precision by the manufacturer for ^13^C is 0.1 per mil.

To extract DNA from hyphae-influenced soil, frozen soil from the no-plant compartment was placed under a dissecting microscope and allowed to briefly thaw to aid manipulation. For +AMF microcosms, 250 mg of soil containing visible hyphae (ca. 2 mm in diameter) was collected using sterile tweezers and weighed into DNeasy PowerSoil bead tubes (Qiagen) and extracted the same day using the PowerSoil kit. For no-AMF controls, the soil was sampled the same way, except no hyphae were visible.

### Hyphosphere HT-SIP density gradient separations

We added 350 ng of ^13^C- and ^12^C-AMF DNA (*n* = 3 each) to density gradients and ultracentrifuged as described above, and then fractionated, precipitated, and quantified using the HT-SIP pipeline. DNA pellets were resuspended in 10 mM Tris-HCl (pH 7.5) because the low DNA mass in each fraction required us to use a large fraction volume during sequence library creation and would have resulted in higher than recommended EDTA concentrations (>0.1 mM EDTA final concentration).

### Metagenomic library preparation and sequencing

We sequenced metagenomes from 14 fractions per SIP gradient. Fractions with low concentrations of DNA at the beginning (fractions 3–6) and end of the gradient (fractions 19–21) were combined and concentrated prior to sequencing using an Amicon Ultra-0.5 30-kDa filter (Millipore Sigma) [53]; fractions 7–18 were sequenced without concentration. Metagenomic libraries were prepared at Lawrence Livermore National Laboratory (LLNL) using the Illumina DNA Flex library kit (now called Illumina DNA Prep, Illumina Inc.) using 1 ng of sample DNA and 12 cycles of amplification. The libraries were dual indexed with Illumina Nextera DNA CD indexes following the manufacturer’s recommended protocol and quantified using a Qubit broad-range dsDNA assay (Thermo Fisher Scientific). Library insert sizes were determined via Agilent Tapestation with the D5000 High Sensitivity assay (Agilent Technologies). Equimolar amounts of each library were pooled together. The size and concentration of the pooled libraries were verified using the D5000 High Sensitivity assay (Agilent Technologies).

The library was diluted and denatured as described [54] to a final concentration of 375 pM (2% phiX and 98% library). The library pools were sequenced with NextSeq 1000/2000 P2 or P3 reagents (Illumina Inc.) as paired end 2 × 150 cycles on an Illumina NextSeq2000 sequencer at LLNL. In total, 2.6 × 10^9^ read pairs passed quality filtering, with a mean of 3.1 × 10^7^ read pairs per sample/fraction and a range of 3.0 × 10^6^ to 1.1 × 10^8^ read pairs.

### Metagenome assembly and annotation

Our prior research indicates that co-assemblies of all SIP gradient fractions from biological replicates yield the best metagenome-assembled genomes (MAGs) [33, 55]. Metagenomic sequences were loaded into IMG v5.1.1. We created individual assemblies using metaspades [56] and a single co-assembly including all fractions using MetaHipMer v2 [55]. MetaHipMer produced more contigs (Supplemental Table S2), so we proceeded with this assembly. Sequences were mapped to the assembly using bbmap [57], binned with Metabat v2.12.1 [58], and then bins were refined with metaWRAP v1.2.1 [59]. MAG quality was determined by CheckM v1.1.3 [60], taxonomy determined by GTDB-tk v1.50 with version r202 of the GTDB taxonomy database [61]. Final metrics are reported using Minimum Information about a Metagenome Amplified Genome (MIMAG) standards [62] (Supplemental Table S3). MAG abundances were determined using BBSplit (BBMap v38.56) [57]. The sequenced genome of *Rhizophagus irregularis* DAOM 197198 was included in the bbsplit reference library to map AMF sequences (RefSeq assembly accession: GCF_000439145.1) [63]. The MAG sequences from this study are deposited under NCBI BioProject accession PRJNA860067.

The genomic capacities of high- and medium-quality MAGs of highly ^13^C enriched hyphosphere taxa were determined by annotating functional categories using Patric subsystems [64] and KEGG orthologs from KofamKOALA [65]. Carbohydrate-active enzyme (CAZy) gene homologs were annotated using dbCAN HMM analysis [66], and potential carbohydrate substrates were assigned for a subset of glycoside hydrolases as per Berlemont and Martiny [67]. Protease homologs were determined using blastp against the MEROPS v.12.1 database (E-value cutoff of 10^−4^) [68]; an initial screen was performed using the “merops_scan.lib” database, and positive hits were then compared to the larger “pepunit.lib” database, which is a non-redundant library of all peptidase units and inhibitor units in MEROPS. Non-proteases (second identifier starts with “9”) and protease inhibitors were removed.

The CAZy gene content from our 30 Myxococcota MAGs were compared to 25 Myxococcota genomes that are GTDB type species and have previously been used for comparative genomic analysis of Myxococcota [69]. MAGs were clustered with the 25 type species (Supplemental Figure S3) using one-dimensional hierarchical clustering, and 7 representative genomes were selected from the major clusters to simplify displaying the data. The NCBI assembly accessions used for the type species are listed in the figure legend of Supplemental Figure S3.

### Quantitative SIP analysis

We used quantitative SIP (qSIP) calculations to estimate the atom percent excess (APE) ^13^C enrichment for each taxon following procedures detailed in Hungate et al. [20] using R statistical software [70], with adjustments for metagenome-assembled genomes instead of 16S rRNA genes [33, 36]. qSIP estimates the APE of each taxon by comparing the density shifts of sequenced reads from enriched samples versus the natural abundance controls. To accomplish this, we converted MAG sequence counts into estimates of DNA mass (ng) by calculating the portion of the sequencing library attributable to the MAG and multiplying by the DNA content per fraction: ([MAG_counts / total_library_counts] * ng_fraction_DNA). Since our reference database of MAGs is incomplete and does not include genomes for eukaryotes and fungi other than the AMF *R. intraradices*, we used this procedure to estimate DNA because the total library counts include organisms with no sequenced representatives. After estimating DNA mass for each MAG, the weighted average density (WAD) of the reads was calculated for each gradient; the difference in WAD between the ^13^C and ^12^C gradients was used to estimate the atom percent excess for each MAG and associated 90% confidence intervals [20]. All MAGs were present in all three replicates. We defined the ^13^C-hyphosphere as all MAGs that were detectably ^13^C-enriched and required the MAGs’ 90% lower confidence intervals be > 0% APE-^13^C.

## Results

### Reproducibility of automated fractionation

**Fig. 1:**
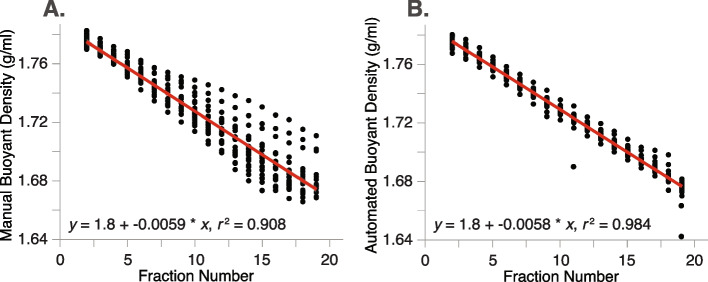
Comparison of manual versus automated fractionation of SIP density gradients. Fractionation is the process of dividing a SIP density gradient into multiple fractions. **A** For “manual” fractionation, 22 independent density gradients were fractionated by visually counting and collecting droplets in microcentrifuge tubes using the method described in Blazewicz et al [17]. Buoyant density (g/ml) for each fraction is measured via refractometry and is represented by a single dot. The number of fractions collected from manual density gradients was variable (range 15–27 fractions per gradient; only fractions 2–19 are displayed). **B** For automated fractionation, 24 independent density gradients were fractionated robotically using an Agilent Technologies fraction collector, which automatically divides the gradients into fractions of a set volume (~236 μl) and dispenses them into a 96-well plate. Automated density gradients consistently produced 22 fractions per gradient (only fractions 2–19 are displayed). Fractions at the beginning and end of the gradient (fractions 1, 20–22) were excluded as these densities are altered by the water used to displace the gradient and are not typically used for molecular analysis

In tests of manual versus HT-SIP procedures, automated fractionation increased the reproducibility of fractionation; the buoyant densities of robotically fractionated samples had a more consistent linear fit across gradients (*r*^2^ = 0.984, n = 24 gradients) compared to manually fractionated gradients (*r*^2^ = 0.908, *n* = 22 gradients) (Fig. 1). Manual fractionation produced a variable number of fractions under identical fractionation conditions (range = 15–27 fractions), which resulted in a wide spread of fraction densities at the lower densities. In contrast, automated fractionation consistently resulted in 22 fractions.

### Effect of non-ionic detergents on automated DNA recovery

Post-fractionation DNA recovery is a key concern for DNA-SIP studies, especially for samples where total DNA mass is limited. We tested how adding the non-ionic detergents Tween-20 and Triton-X affected DNA recovery in density gradients, which can reduce potential DNA adsorption to polypropylene tube walls [51]. Adding Tween-20 at final concentrations ≤ 0.01% reliably increased the recovery of 1 μg pure culture *E. coli* DNA (Supplemental Table S1), and Tween-20 at 0.0001% yielded the highest overall DNA recovery (84 ± 5%, 95% CI) compared to a no-detergent control (64 ± 5%, 95% CI) (Student’s *t* test, *p* = 0.004). Triton-X only improved DNA recovery at a final concentration of 0.0001% (74 ± 3%, 95% CI) (Student’s *t* test, *p* = 0.02).

**Fig. 2:**
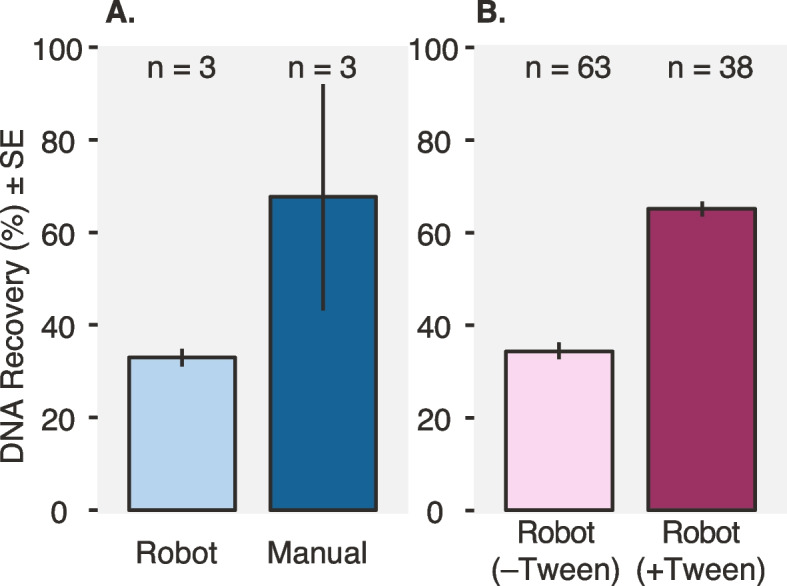
DNA recovery comparison for manual and semi-automated PEG precipitation methods, and the impact of adding a non-ionic detergent (Tween-20) to the SIP gradient buffer. After a density gradient is fractionated, DNA from each fraction needs to be purified (desalted) prior to quantification and sequencing analysis. **A** We compared “manual” PEG precipitations of soil DNA (*n*=3 SIP gradients), where each fraction is precipitated in microcentrifuge tubes by an individual (as per Blazewicz et al. [17]), and semi-automated or “robot” PEG precipitations (*n*=3 SIP gradients), where a Hamilton STAR liquid handling robot performs the precipitations in 96-well plates. **B** In a separate study, we tested how adding Tween-20 to the density gradient mixture impacts DNA recovery for a large soil DNA-SIP experiment; all samples were processed semi-automatically by the robot. Tween-20 was added to a subset of the samples (+Tween, *n* = 38 SIP gradients) or processed using our standard density gradient buffer without Tween-20 (–Tween, *n* = 63 SIP gradients). Four micrograms soil DNA was used per SIP gradient, and percent DNA recovery was calculated by summing recovered DNA (measured by Picogreen) across all density fractions post-cleanup and dividing by the initial DNA input. Error bars represent the standard error of the mean

Using soil DNA in the absence of Tween-20, following precipitation, we had higher DNA recovery with manual processing relative to the automated protocol (Fig. 2A). However, when 0.0001% Tween-20 was included in the density gradient buffer, soil DNA recovery with the automated HT-SIP protocol was comparable to the manual protocol (Fig. 2B). For the automated protocol, adding Tween-20 significantly increased soil DNA yields by 1.8-fold from 35 ± 2% to 64 ± 2% (mean ± SE; Student’s *t* test, *p* < 0.001).

### SIP automation time savings

HT-SIP automated processing had significant time savings and substantially decreased the amount of manual or “hands-on” labor required for SIP. HT-SIP required half the technician time to fully process 16 samples (42 h versus 20 h for 16 samples), and one-sixth the hands-on hours compared to serially processing single samples manually (Table 1); within a work week, this represents 3.2 days of hands-on labor saved. Using automation, it is manageable for a single technician to process 16 samples in parallel (this batch number is constrained by the typical number of spaces available in an ultracentrifuge rotor). Manual estimates assume no parallelization and are based on the minimum amount of time required to process a single SIP gradient under ideal conditions in our laboratory.

**Table 1:** Time comparison for manual versus automated SIP fractionation, cleanup, and DNA quantification. Estimates are based on processing 22 fractions per SIP tube. The “hands-on” columns indicate the time an individual must actively manipulate the samples, while “total” columns indicate the total time required for the entire process. Time estimates for manual cleanup and quantification are based on processing a single SIP gradient and assume maximum processing speed. Automated fraction cleanup time is based on the time required to fractionate a single tube using the Agilent Infinity Fraction Collector. Automated sample cleanup and DNA quantification times are based on the processing times for the Hamilton STAR liquid handling robots. The † symbol indicates that 16 SIP gradients are processed simultaneously in 4 plates (4 gradients per plate), and the “per 1 sample” times are calculated by dividing the total time by 16. “Batch” processing times are the times required to process 16 density gradients

SIP processing steps	Manual time (hours)		Automated time (hours)	
	Hands-on	Total	Hands-on	Total
Fraction collection	1	1	0.2	0.6
Sample cleanup	0.4	1	0.04†	0.5†
DNA quantification	0.5	0.6	0.06†	0.13†
Total time (per 1 sample)	1.9	2.6	0.3†	1.2†
**Batch time (per 16 samples)**	**30**	**42**	**4.8**	**20**

### HT-SIP application: hyphosphere soil

We applied the HT-SIP pipeline to an important yet challenging sample set (low DNA, low overall ^13^C-enrichment): AMF hyphosphere soil. After 6 weeks of ^13^CO_2_ labeling, roots in the plant compartment of ^13^C-AMF microcosms were highly enriched (41.3 ± 1.9 atom% ^13^C). Root biomass in the ^12^C microcosms was unenriched (1.1 ± 0.001 atom% ^13^C). In the ^13^C-AMF microcosms, IRMS analysis of the no-plant compartment showed the soil was slightly enriched (1.8 ± 0.1% atom% ^13^C), whereas in the ^13^C no-AMF control microcosms, the isotopic enrichment of the no-plant compartment was not statistically different from the ^12^C control soil (1.1 ± 0.001% versus 1.1 ± 0.0003%, respectively). This indicates that even though the compartments were not gas tight, ^13^CO_2_ diffusion into the soil from the labeling chamber headspace was low and did not support significant autotrophic ^13^C fixation, and that the enriched ^13^C detected in the no-plant compartment was transferred primarily by the AMF hyphae.

**Fig. 3:**
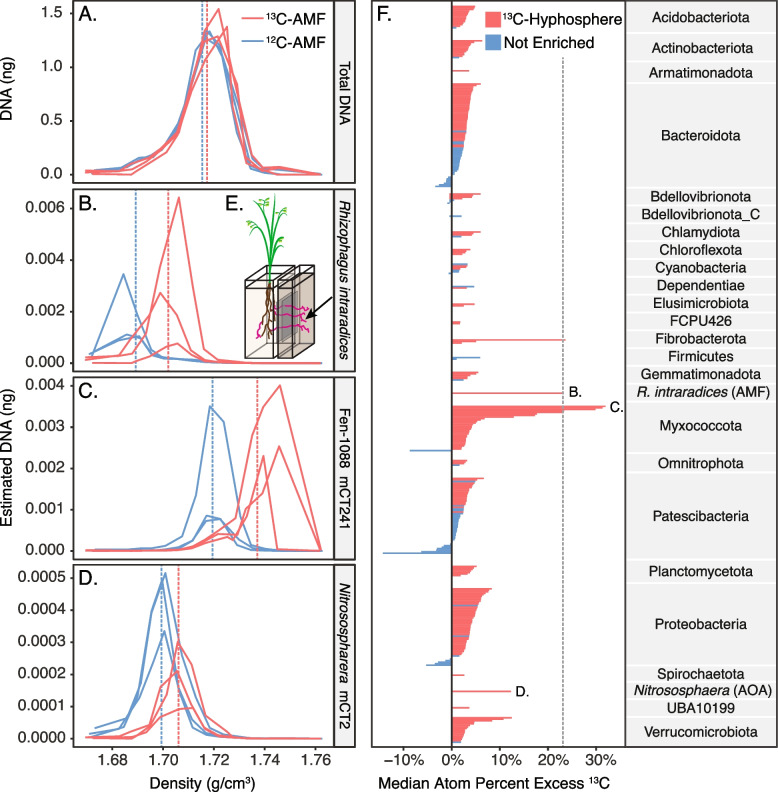
^13^C-Hyphosphere metagenome-assembled genomes (MAGs) isolated by SIP metagenomics. Fraction DNA mass (ng) versus fraction density for **A** total DNA that was fractionated from ^13^C-AMF DNA (red lines) and ^12^C-AMF control DNA (blue lines); *n*=3. Dashed lines are the weighted average density (WAD) of the DNA from the ^13^C and ^12^C replicate gradients. Within each gradient, we used qSIP to estimate taxon-specific DNA masses for **B** the AMF host *Rhizophagus intraradices*, and all MAGs. **C** A Myxococcota MAG (Fen-1088 mCT241) was the most ^13^C-enriched MAG detected, and **D** an archaeal ammonia oxidizer (*Nitrososphaera* mCT2) was among the most ^13^C-enriched MAGs detected. Taxon-specific DNA masses were estimated by multiplying a fraction’s total DNA mass by taxon relative abundance (i.e., MAG counts divided by metagenomic library counts). **E** To isolate hyphosphere soil from the influence of roots, we used a two-compartment experimental microcosm where *Avena barbata* inoculated with AMF grew in one compartment, and AMF hyphae (pink lines) crossed an air gap to access soil in the no-plant compartment; soil aggregates with visible hyphae were collected from this compartment for SIP (see Supplemental Figure S2 for detailed experimental design). **F** We calculated the estimated median atom percent excess (APE) for all assembled MAGs, where red bars indicate the 212 ^13^C-hyphosphere MAGs with detectable isotopic enrichment (lower 90% CI bound greater than 0% APE-^13^C), and blue bars indicate 87 bulk soil MAGs that were unenriched (lower 90% CI bound below 0% APE-^13^C); error bars are suppressed for readability. MAGs are grouped by phylum, and letters indicate the APE of the taxa shown in panels **B–D**. The dashed gray line indicates the APE of the AMF, which supplied ^13^C to the no-plant compartment. Inset diagram (**E**) modified with permission from Kakouridis et al. [41]

To identify ^13^C-enriched metagenome-assembled genomes (MAGs) in the hyphosphere, we used the HT-SIP pipeline on DNA extracted from hyphae-influenced soil collected from the no-plant compartment in both ^13^C- and ^12^C-AMF microcosms. Initial density separations of total DNA showed evidence of only slight ^13^C-enrichment, as seen by the small increase in weighted average density (WAD) between ^12^C and ^13^C samples (Fig. 3A). After mapping the SIP metagenomic reads to the *R. irregularis* genome, we calculated APE using qSIP and found that the AMF DNA was highly ^13^C-enriched at 23 atom% ^13^C (Fig. 3B).

### Hyphosphere-qSIP metagenome assembly and binning

All fractions were co-assembled using MetaHipMer2, which assembled 14.4 Gbp > 1 kb and 1.6 Gbp > 10 kb. Compared to single fraction assembly using metaspades, MetaHipMer2 produced 3.3 and 3.2 times more assembled contigs >1 kb and >10 kb, respectively (Supplemental Table S2). We therefore proceeded with the MetaHipMer2 assembly (which did not require bin dereplication). Overall, 71.2% of the sequence reads mapped to the MetaHipMer2 assembly. Binning produced 299 medium- and high-quality MAGs; completeness, percent contamination, and MAG genome size are available in Supplemental Table S3. Three MAGs were 100% complete (determined by CheckM) and were from the following orders: Cytophagales (mCT1; Bacteroidota (Bacteria); 1.2% contamination), Nitrososphaerales (mCT2; Thermoproteota (Archaea); 2.9% contamination), and Pedosphaerales (mCT3; Verrucomicrobiota (Bacteria); 4.9% contamination). The phyla with the most MAGs assembled were Bacteroidota (68 MAGs), Proteobacteria (51 MAGs), the candidate phylum radiation Patescibacteria superphylum (50 MAGs), and Myxococcota (30 MAGs). On average, 13.3 ± 7.6% SD of the sequence reads mapped to the MAGs.

### Genomic potential of ^13^C-enriched MAGs in the AMF hyphosphere

**Fig. 4:**
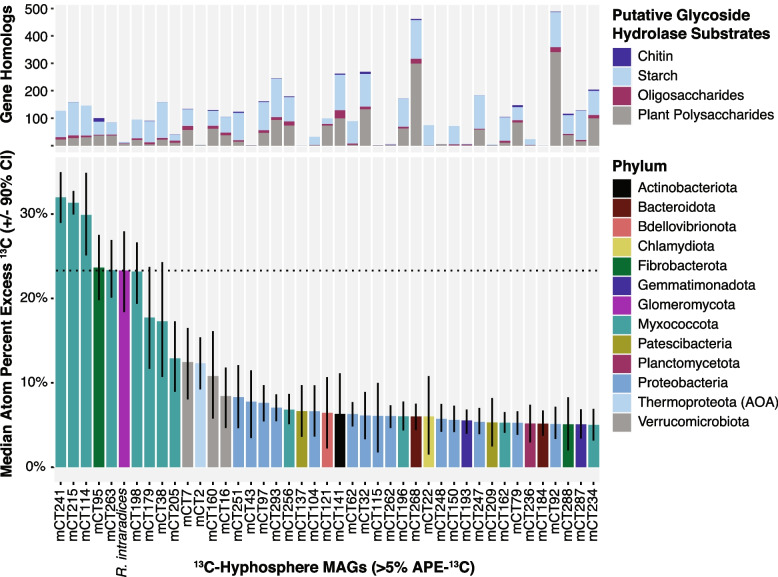
Highly and moderately ^13^C-enriched MAGs in the AMF hyphosphere and their CAZy genomic potential. MAGs displayed have >5 atom percent excess (APE) ^13^C and are colored by phylum affiliation in the bottom panel. The top panel indicates putative substrates of select glycoside hydrolase CAZymes gene homologs for each MAG (potentially targeting chitin, starch, oligosaccharides, and plant polysaccharides, as per Berlemont and Martiny [67]). The dashed black line indicates the APE-^13^C of the AMF, *Rhizophagus intraradices*. Error bars represent the 90% confidence interval. A full list of all MAGs and their isotopic enrichment is available in Supplemental Table 3

The soil microbial community in the ^13^C AMF hyphosphere was isotopically enriched; of the 299 assembled MAGs, 212 were detectably ^13^C enriched, indicating they consumed plant-fixed ^13^C transported by the AMF hyphae (Fig. 3E). Of these, 43 MAGs were moderately enriched (> 5–10% APE-^13^C) and 12 MAGs were highly enriched (>10% APE-^13^C). Highly enriched MAGs included 8 Myxococcota, one Fibrobacterota from the family UBA11236 (mCT95), one ammonia-oxidizing archaeon (AOA) from the genus *Nitrososphaera* (mCT2), and two MAGs from the Verrucomicrobiota family Opitutaceae (mCT7, mCT160) (Fig. 4).

We used comparative genomics of carbohydrate degradation genes (CAZymes), as well as other genes, to assess possible roles for these MAGs in the hyphosphere food web based on their genomic potential. Except for the AOA, highly ^13^C-enriched MAGs had multiple homologs of PL6 genes (alginate and non-alginate polysaccharide lyases [57]) and GH109 genes (glycoprotein α-N-acetylgalactosaminidases) (Supplemental Table S4); PL6 family enzymes were disproportionately abundant in highly ^13^C-enriched MAGs (11 of the 29 total MAGs containing PL6).

**Fig. 5:**
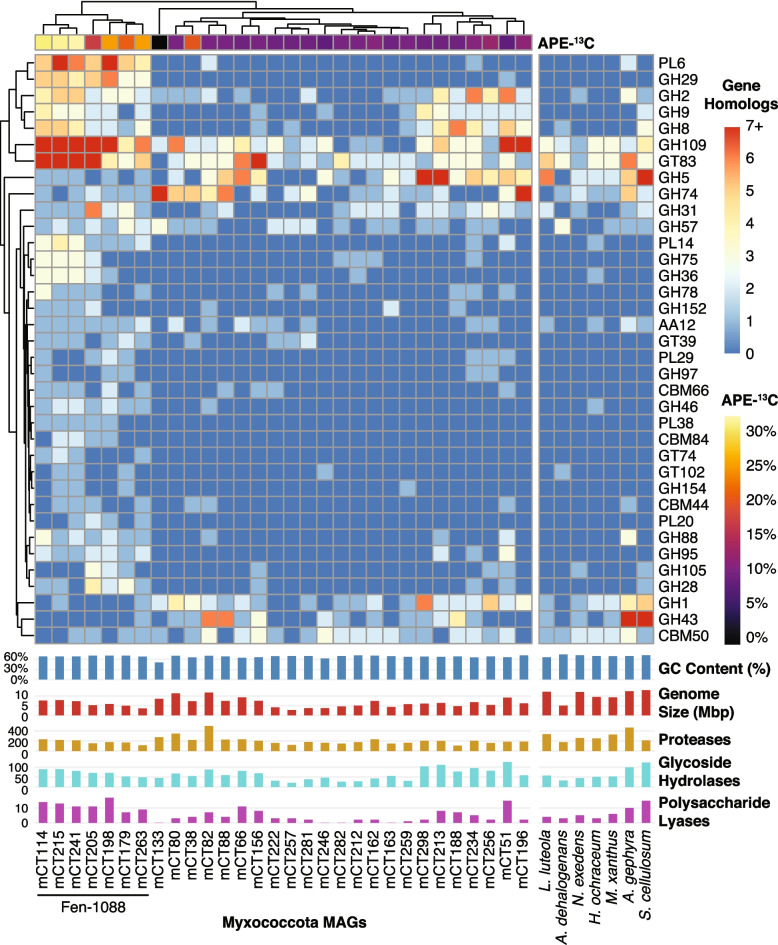
Genomic comparison of Myxococcota MAGs versus Myxococcota type species for CAZyme gene content and genome characteristics. Heatmap rows indicate the number of gene homologs (red-blue color scale) detected per CAZy family, and columns indicate the associated MAGs. Atom percent excess ^13^C (APE-^13^C) estimated by qSIP is presented in the top row (yellow-purple-black color scale). Columns and rows were grouped using one-dimensional hierarchical clustering based on genomic content. CAZy families displayed had significantly more or less genes detected in the Fen-1088 MAGs compared to the rest of our Myxococcota MAGs (Student’s *t* test, *p* <0.05); full CAZy results for the MAGs are available in Supplemental Table S6. Bar graphs indicate genome %GC content and genome size (Mbp), as well as number of protease, glycoside hydrolase, and polysaccharide lyase gene homologs per genome. The type species (*Labilithrix luteola*, *Anaeromyxobacter dehalogenans* 2CP-1, *Nannocystis exedens*, *Haliangium ochraceum* DSM 14365, *Myxococcus xanthus*, *Archangium gephyra*, *Sorangium cellulosum* B) were clustered with our MAGs and 25 other Myxococcota type species (Supplemental Figure S3); a single type species genome was selected the represent each cluster for this figure. Acronyms: GH = glycoside hydrolase; PL = polysaccharide lyase; CBM = carbohydrate binding module; GT = glycosyltransferase; AA = auxiliary activity

Eight MAGs from the phylum Myxococcota were among the most ^13^C-enriched taxa detected (12–32% APE-^13^C), and seven of these were from the little-known Polyangia family Fen-1088 that is known only from metagenomic sequencing (only 31 MAGs in the Genome Taxonomy Database (GTDB), accessed June 2022) [71]. Similar to many Myxococcota [69], Fen-1088 have high GC contents (68–71%) and large genome sizes (3.5–7.8 Mbp), though these are smaller than the largest Myxococcota genomes (Fig. 5). Three highly enriched Fen-1088 MAGs (mCT241, mCT215, mCT114) were ca. 8% APE-^13^C more enriched than the AMF and ranged from 30 to 32% APE-^13^C (Fig. 3C, mCT241). Compared to a set of Myxococcota type species genomes, these highly enriched MAGs had many glycoside hydrolases and polysaccharide lyases that are atypical for Myxococcota type species (Fig. 5) (Supplemental Figure S3), such as GH29 (α-fucosidases), GH109 (α-N-acetylgalactosaminidases), GH8 (β-1,4 linkages, such as those found in plant cell walls), PL6 (alginate and non-alginate lyases [72]), and PL14 (lyases of unknown function, including poly(b-mannuronate) lyase). These genomic differences are also apparent across the Myxococcota MAGs from this study, where the highly ^13^C-enriched Fen-1088 MAGs consistently contained more PL6 and GH29 homologs than less-enriched Myxococcota (Fig. 5). All Myxococcota MAGs contain many proteases (Supplemental Table S5), and the three most enriched Fen-1088 MAGs contain 203-225 MEROPS protease homologs (Fig. 5). Only two of the Fen-1088 MAGs contain a chitinase gene (mCT114, mCT215) (Supplemental Table S6).

Fibrobacterota mCT95 had the same ^13^C-enrichment as the AMF (23% APE-^13^C) and has chitinolytic potential (9 genes with GH18 domains and 2 genes with GH19 domains, 6 of which were annotated as chitinases (EC 3.2.1.14)). This Fibrobacterota MAG also contains GH74 genes for lysing β-1,4 glucan linkages in plant cell wall polysaccharides.

The AOA *Nitrososphaera* mCT2 MAG (Fig. 3D) was estimated to be 100% complete and enriched at 12% APE-^13^C. This MAG contains genes for ammonia oxidation (amoABC) and nitrite reductase (nirK), as well as the marker gene for the 3-hydroxypropionate/4-hydroxybutyrate cycle for autotrophic C fixation (hydroxybuteryl-CoA dehydratase) [73]. *Nitrososphaera* mCT2 also has a urease gene cluster with a complete set of urease structural genes (ureA, ureB, ureC) and a urease accessory gene (ureG). In terms of CAZy gene content, *Nitrososphaera* mCT2 has low glycoside hydrolase content (only 7 genes), no polysaccharide lyase genes, but many glycosyltransferase genes (24 genes).

The Verrucomicrobiota MAGs from the Opitutaceae (mCT7, mCT160) were 11–12% APE-^13^C enriched. These genomes contain multiple genes with CAZy domains for processing glucose- and galactose-based uronic acids, such as genes for lysing β-1,4 linkages in polyglucuronic acid (PL20), hydrolyzing α-1,4 glycosidic linkages in polygalacturonic acid (GH28), and hydrolyzing glucuronic and galacturonic acid monomers (GH105). Polygalacturonases lyse the pectin in plant cell walls [74], while polyglucuronases target the cell walls of bacteria, fungi, and algae [75]. Compared to the other highly enriched MAGs, the Opitutaceae MAGs contain more genes that could potentially depolymerize plant polysaccharides (e.g., cellulase), but the MAGs with the largest sets of potential plant decomposition genes were medium-enriched (5–10% APE-^13^C) (Fig. 4, top panel) and low-enriched MAGs (< 5% APE-^13^C) (Supplemental Table S7). Similar to the Myxococcota MAGs, the Opitutaceae MAGs also contain multiple copies of GH29 α-fucosidases. A detailed list of taxonomy and isotopic enrichment for all MAGs is available in Supplemental Table S3, and a comparison of the CAZyme gene content for MAGs with >10% APE-^13^C is available in Supplemental Table S4.

## Discussion

Stable-isotope probing is a powerful technique for resolving population demographics, ecophysiology, and interactions between active microorganisms in situ, without the need for cultivation. However, SIP is not as broadly used as it could be in part because it is relatively low throughput, time-consuming, and highly manual. We have designed a high-throughput semi-automated SIP pipeline, “HT-SIP,” which enables substantially higher sample throughput, reduces labor demands, and makes it more feasible to invest effort in high-risk low-biomass samples, such as the AMF hyphosphere samples analyzed in this study. Using HT-SIP, we were able to target an important microhabitat, the AMF hyphosphere, and examine potential trophic interactions in the fungal hyphosphere based on ^13^C-enrichment.

### SIP DNA recovery

The amount of DNA recovered in each SIP density fraction can be a limiting factor for library construction. Adsorption of DNA to polypropylene tube walls can potentially lead to substantial sample loss, especially when DNA is in a high ionic strength solution [51], such as CsCl gradient buffer. Adding a low concentration of Tween-20 (0.0001%) led to a near doubling of DNA yield. Using this method, samples with only 1 μg DNA and below (such as our hyphosphere soils) can be more reliably processed and analyzed using metagenomic sequencing technologies that have low DNA input requirements. However, we recommend adding 3–5 μg DNA if conducting multiple analyses, so that 100 ng DNA may be recovered per fraction and provide 20 ng DNA for three analyses: metagenomic sequencing, 16S rRNA gene and ITS amplicon libraries, and qPCR assays. Here, we successfully analyzed 350 ng DNA per hyphosphere DNA sample with 42% DNA recovery, which was suitable for low-input metagenomic sequencing of the fractions containing DNA.

### HT-SIP optimization

Automating the SIP process significantly decreased the required operator hours (Table 1) while simultaneously improving reproducibility. Traditionally, after manually fractionating a density gradient, an additional 1–2 h per sample is required to desalt the gradient fractions (nucleic acid purification). Thus, in many research groups, a maximum of 6–8 samples may be processed per week—a grueling prospect for analyzing large experiments if all work must be completed by hand. HT-SIP makes it possible to routinely process 16 samples per week, since the overall time to process samples is decreased by half and laborious “hands-on” tasks are decreased by one-sixth. These time savings translate into substantial labor cost savings, which over time can offset the initial sizable cost of purchasing robotic instruments. The cost of supplies and reagents is similar for the robotic and manual approaches.

We also tested other optimization techniques that were not adopted as part of our final pipeline. We attempted magbead cleanups to desalt the CsCl gradient fractions (Ampure XP, Beckman Coulter); in our hands, we found that PEG precipitations had higher yields. Others have found that molecular weight size exclusion filters are an effective alternative for CsCl desalting [9, 76]. We also assessed if DNA intercalators could be used to minimize the need for separate ^12^C controls. Actinomycin D reduces the native buoyant density of GC-rich DNA [77] and theoretically reduces 90% of natural DNA density differences. However, we found that the high concentrations needed to reduce the density of GC-rich DNA (>10 μg actinomycin D incubated for 10 min) also reduced overall DNA recovery. While we did not pursue further use of intercalators, with some optimization this could be a viable approach.

### Processing hyphosphere-SIP samples with low isotopic enrichment

Assessing a sample’s isotopic enrichment (e.g., via mass spectrometry) is often conducted as a pre-screen when deciding whether to proceed with SIP density gradient separations. Based on our bulk soil IRMS data and the poor separation between DNA density curves (Fig. 3A), we typically would not have proceeded with our ^13^C-SIP AMF study because the isotopic enrichment appeared to be too low. However, since AMF are obligate biotrophs and the plant biomass was highly ^13^C enriched, we hypothesized that AMF hyphae and their hyphosphere were also highly enriched, and that the low bulk ^13^C value might have been caused by dilution from ^12^C bulk soil collected with the hyphae. To increase the chance of detecting enriched taxa, we proceeded with a DNA-SIP strategy of sequencing all DNA-containing fractions (14 total), because samples with lower isotopic enrichment require more replicates and fractions to detect taxon-specific enrichment [48]. While initial IRMS data and DNA density curves suggested SIP might be impracticable due to insufficient ^13^C label, instead, taxon-specific qSIP indicated a highly enriched ^13^C-AMF signal and identified 212 ^13^C-enriched hyphosphere MAGs. The large number of demonstrably enriched MAGs suggests that future hyphosphere-SIP studies could potentially occur over shorter incubation periods (< 6 weeks), which would help identify early incorporators of AMF-C and reduce cross-feeding within the microbial food web [19].

### Dissecting trophic interactions in the AMF hyphosphere

AMF serve important roles in the soil microbial food web by stimulating soil organic matter decomposition [42–44] and providing recently fixed plant C to the soil community [78]. Paradoxically, AMF are capable of stimulating decomposition of SOM and detritus [39, 40], but do not have the enzymatic repertoire to decompose SOM themselves. We and others have hypothesized that AMF collaborate with their soil microbiome to mineralize organic nutrients [45]; previous research shows the presence of AMF modifies the litter-decomposing microbiome [40] and the hyphosphere microbiome can help acquire organic N that is then transferred back to the plant host [79]. In addition to stimulating the decomposition of detrital organic matter, AMF also rapidly distribute a significant portion of newly fixed photosynthate C into the soil system [78]; this C is then distributed to a larger volume of soil than what is accessible by roots, as AMF hyphae are estimated explore two orders of magnitude more pore volume than plant roots [80].

The ^13^C-AMF hyphosphere MAGs we identified in this study are a key advance, providing a molecular resource that will enable future experiments studying how AMF alter decomposition and carbon cycling trophic processes and determining the molecular mechanisms that underpin them. Because our SIP metagenomes targeted DNA from actively growing hyphosphere organisms that consumed ^13^C substrates or ^13^CO_2_ derived from AMF, the genomic content of these MAGs represents a crucial part of the hyphosphere microbial food web and provides a means to dissect potential trophic interactions—such as predation, organic matter decomposition, and ammonia oxidation.

### Predation in the hyphosphere

Using qSIP-estimated MAG atom percent excess (APE), we examined potential trophic interactions in the fungal hyphosphere based on ^13^C-enrichment. Intriguingly, Myxococcota from the poorly characterized family Fen-1088 were the most enriched taxa detected in the hyphosphere. While Myxococcota are a known component of AMF hyphosphere communities [81], their functional role has not been previously determined. The particularly high atom percent ^13^C enrichment of the Fen-1088 family in our dataset suggests they are either directly feeding upon C exuded by the AMF, or they are predators which target AMF hyphae or other microbes in the AMF hyphosphere. The Myxococcota phylum contains many facultative predators that can subsist by consuming microbial or plant organic matter [82] and have a broad prey range including bacteria and fungi [83, 84]. Since our Myxococcota MAGs have high GC content and large genomes, it is unlikely that they are AMF endosymbionts (which often have reduced genomes). Previous GWAS analysis indicates that Myxococcota have many prey-specific genes, rather than a general set of antimicrobial genes, which likely enable them to target a broad prey range [85]. Our analysis of the Fen-1088 MAGs also points to a large arsenal of proteases that may be used to consume prey; these are also found in many Myxococcota type species [69]. Proteases depolymerize proteins into peptides, amino acids, and eventually ammonium, and are key to the creation of bioavailable N [86]. However, the relative lack of GH18 chitinase genes suggests Fen-1088 may not be chitinolytic or may have low chitin degradation efficiency, as highly efficient chitinolytic organisms are thought to produce multiple types of chitinases [87, 88]. Organisms other than Fen-1088 may instead be performing this role in the microbial food web; for example, the highly ^13^C-enriched Fibrobacterota mCT95 has numerous chitinases which suggests it has high chitinolytic potential.

While the Fen-1088 MAGs are similar to other Myxococcota genomes (with a large array of proteases and high GC content), they also contain multiple copies of genes with CAZyme domains that are uncommon in Myxococcota type species. Most of these CAZymes are hypothetical proteins, but the presence of these CAZyme domains hints at potential function. We consistently found two enzyme groups in Fen-1088 MAGs: GH29 and PL6. The GH29 group is known to contain alpha-fucosidases, which remove terminal L-fucoses from oligosaccharides or their conjugates [89]. Many biomolecules are fucosylated [90]—polysaccharides, glycoproteins, and glycolipids can have attached fucoses [89]. Fucose can be exuded and tightly attached to polysaccharides the AMF hyphal surface [91], and AMF exude fucosylated lipo-chitooligosaccharides that can act as signaling molecules when establishing a symbiosis with a plant host [92, 93]. Much less is known about polyspecific enzyme family PL6; these enzymes are abundant in Fen-1088 and our MAGs with >10% APE-^13^C (except the AOA). PL6 contains alginate lyases and non-alginate lyases [72] that cleave ß (1-4) linkages within polysaccharides built from mannuronic and guluronic acids [72], such as alginate produced by brown algae and some bacteria [94], or between these acids and other building blocks for non-alginates. Mannose, the sugar from which mannuronic acid is built, is abundant in the mono- and polysaccharides bound to the surface of AMF hyphae [91], but we have no information about how these monosaccharides are polymerized. Further research on both the polysaccharides in the AMF hyphosphere and the feeding preferences of the Fen-1088 Myxococcota are needed to determine the nature of their relationship with AMF.

Myxococcota were some of the most enriched MAGs detected in this study, which may be because they are acting as predators in the hyphosphere food web. Stable isotope food web studies indicate that the ^13^C enrichment of an organism is a conservative indicator of the ^13^C enrichment of the substrate they consumed [31, 95]. A recent qSIP meta-analysis showed that predators assimilate ^18^O or ^13^C at higher rates than non-predators [31], which allows them to achieve high levels of isotopic enrichment in a short period of time relative to non-predators—within the bounds that an organism’s isotopic enrichment is ultimately limited by the substrate they consume. Another qSIP study found that viral DNA was more ^13^C-enriched than its microbial host, likely because viruses replicate rapidly and create new DNA inside the cell, which allows their enrichment to quickly match that of the substrate consumed by the host [36]. We observed that the Fen-1088 MAGs were actually more ^13^C enriched than the AMF, which suggests that the Myxococcota were likely consuming ^13^C-rich compounds recently transported by the AMF from the plant host, such as newer hyphae or fresh exudates. This is because, at the time of harvest, the bulk ^13^C-enrichment of the plant roots was 41 atom% (IRMS) and AMF hyphae were 23 atom% (estimated by qSIP, which averages across all AMF biomass); we expect that recently produced hyphae and exudates had a higher isotopic signature more similar to the plant host. We note that isotopic concentration by predators due to isotopic fractionation (ca. 1 per mil per trophic level for C [95]) is not relevant to our study, since we added ^13^C as a tracer and the system was highly enriched in ^13^C.

### Interactions between AMF and potential decomposers of fungal necromass or plant detritus

The depolymerization of macromolecules that make up fungal and plant biomass is a major rate-limiting step in C and N cycling and regulates the disassembly of polymers into bioavailable forms, such as monomers [86]. AMF hyphae turnover quickly (5–6 days [96]), and cycling this necromass represents a potentially rapid flow of nutrients and photosynthate C into a large volume of the soil system [45, 80]. Recycling AMF necromass for C and N is likely a key nutrient flow in the soil microbial food web [45], and necromass depolymerization could potentially recycle nutrients contained within decaying hyphae and make them available again to the AMF. Almost all of our study’s highly ^13^C-enriched MAGs (except the AOA) have the genomic potential to degrade AMF fungal biomass. These taxa contain GH109 genes, whose primary reported activity to date is α-N-acetylgalactosaminidase targeting glycoproteins. Polysaccharides on the surface of AMF hyphae contain galactose [91], and lectin-binding analysis indicates AMF contain galactose-based glycoproteins [97]. AMF hyphae have also previously been correlated with citrate-extractable glycoproteins (and related compounds) that may play a role in soil aggregation and C stabilization [98]. Another component of fungal biomass is chitin, which is an abundant macromolecule that contains N [87, 99]. Depolymerization of AMF chitin to chitooligomers, N-acetylglucosamine, and eventually ammonium could be an important source of N in the hyphosphere, and AMF were recently shown to stimulate efficient recycling of exogenously applied ^15^N-chitin [100]. One of the most enriched MAGs, Fibrobacterota mCT95, has 11 putative chitinase genes (GH18, GH19) and has the same isotopic enrichment as the AMF. Thus, the Fibrobacterota is likely deriving most of its C from the AMF and could be performing a chitinolytic function in this food web.

Almost all the highly enriched MAGs and many of the low- to medium-enriched MAGs have enzymatic potential to depolymerize plant biomass. The Fibrobacterota and Verrucomicrobiota family Opitutaceae MAGs have isolated relatives that are thought to be involved in soil organic matter decomposition. Fibrobacterota include cellulose degrading bacteria found in mammal rumens [101], termite guts [102], anaerobic cellulose reactors [103], and rice paddy soil [104]. The family Opitutaceae contains isolates derived from rice paddies and insect guts [105–108]. In our previous study of decomposition gene expression in the rhizosphere and detritusphere using metatranscriptomics, we found that Fibrobacterota and Opitutaceae were two of the three groups that exhibited the highest decomposition gene expression when both root exudates and detritus were available [109]. Further examination would be required to determine if the MAGs from this study exhibit similar synergistic behavior in the AMF hyphosphere, and whether this stimulates the decomposition of plant residues.

### Cross-kingdom interactions between AMF, ammonia-oxidizing archaea (AOA), and soil bacteria

Cross-kingdom interactions, either indirect or direct, are likely responsible for the archaeal MAG *Nitrososphaera* mCT2 becoming one of the most highly ^13^C-enriched organisms detected within our ^13^C-hyphosphere community (12% APE ^13^C). AOA are generally thought to be autotrophic [110], which suggests that the AOA likely acquired ^13^C by fixing ^13^CO_2_ respired by either the AMF or nearby biota that had obtained substantial quantities of ^13^C from the AMF (e.g., Myxococcota, Fibrobacterota*,* Opitutaceae bacteria); however, there is evidence that some AOA isolates can use simple organic compounds (e.g., urea) to supplement energy generation [111–114]. *Nitrososphaera* mCT2 contains a complete set of urease structural genes and a urease accessory gene, which indicates this organism may have the capacity to hydrolyze urea (an organic N monomer) and could have taken up some amount of ^13^C through this route [115]. Urease converts urea into CO_2_ and two ammonium ions (EC 3.5.1.5) and may support AOA nitrification in soil [111, 116]; these enzymes have previously been found to be prevalent in surface soil and subsurface AOA [73, 117]. Because we did not detect ^13^C enrichment in the hyphae-free control soil by IRMS, it is unlikely that the AOA non-specifically fixed external ^13^CO_2_ gas that diffused into the microcosm from the labeling chamber headspace. Therefore, we conclude that AMF played a key role in the transport of plant photosynthate C to the AOA—either through an indirect pathway (AMF ➜ other biota ➜ AOA), or a direct pathway (via the use of exuded simple organic compounds or via fixation of ^13^CO_2_ respired by AMF, AMF ➜ AOA).

Since both AOA and AMF have little to no capacity for saprotrophy, these two groups have been hypothesized to compete for N because they both use ammonium and are limited in their ability to acquire N directly through organic matter depolymerization [118]. AOA generate energy by oxidizing ammonia to nitrite, which is the first and rate-limiting step of nitrification [119, 120]. AMF, on the other hand, are thought to obtain N produced by the soil microbial community [121], such as ammonium or nitrate [122, 123] and possibly organic N monomers [124, 125]. Some of the ^13^C-enriched bacterial MAGs found in this study could potentially produce ammonium and N monomers following organic matter depolymerization, such as MAGs that contain large numbers of proteases (e.g., Myxococcota) or chitinases (e.g., Fibrobacterota). Whether or not AMF and AOA compete for these N sources is an open area of research; studies have shown AMF can have positive, negative, and negligible effects on AOA abundance and activity [126–130]. The high AOA ^13^C-enrichment that we observed indicates that AOA were actively replicating in the hyphosphere and likely contributing to N cycling via ammonia oxidation, though the magnitude of this contribution needs to be determined. Since AOA play an important role in the first step of nitrification [119, 120], cross-kingdom interactions between AMF, AOA, and the soil microbiome have implications for terrestrial N cycling and N_2_O emissions and warrant further research.

## Conclusions

Increasing the throughput of SIP is a crucial step in promoting the well-replicated, temporally resolved experiments needed to study dynamic microbial community activities over space and time. Decreasing the hands-on labor needed to process SIP samples also makes analyzing high-risk samples more feasible, such as the AMF hyphosphere samples analyzed in this study, which had limited soil volume, low bulk isotopic enrichment, and minimal separation based on total DNA density curves. The highly ^13^C-enriched MAGs we identified highlight the potential for cross-kingdom trophic interactions in the AMF hyphosphere, including predation, decomposition of fungal necromass or plant detritus, and archaeal ammonia oxidation that may utilize ammonium or CO_2_ released from the aforementioned processes. In combination with other ‘-omics technologies, such as metatranscriptomics or proteomics, these MAGs will provide an important genomic resource for future experiments exploring interactions between AMF and their native microbiome.

## Supplementary Information

**Additional file 1: Supplemental Figure S1.** Automated fractionation system design, parts, and assembly. **Supplemental Figure S2.** Hyphosphere-SIP experimental design. **Supplemental Figure S3.** Myxococcota MAG CAZymes grouped with CAZymes from 25 genomes from Myxococcota GTDB type species (previously used in Murphy et al. [2]).

**Additional file 2: Supplemental Table S1.** Impact of adding different concentrations of non-ionic detergents to the SIP gradient medium on percent DNA recovery (n=3).

**Additional file 3: Supplemental Table S2.** Assembly metrics using single fraction assembly (metaspades) verses co-assembly of all fractions (MetaHipMer2).

**Additional file 4: Supplemental Table S3.** Assembly statistics, taxonomic lineage, and atom percent excess (APE) of the metagenome amplified genomes (MAGs) created in this study.

**Additional file 5: Supplemental Table S4.** Number of CAZyme family domains in the highly enriched MAGs (>10 atom% excess 13C). AOA = archaeal ammonia oxidizer, Fibro = Fibrobacterota, Myxo = Myxococcota (Myxococcaceae).

**Additional file 6: Supplemental Table S5.** Genomic abundance of MEROPS proteae gene per MAG. Columns indicate MEROPS protease families. APE = atom percent excess.

**Additional file 7: Supplemental Table S6.** CAZymes for the Myxococcota MAGs. Fen-1088 MAGs are highlighted in yellow. MAGs are sorted by increasing atom percent excess (APE) 13C.

**Additional file 8: Supplemental Table S7.** Number of CAZy gene homologs that can potentially target a particular substrate. Substrate assignments based off of the Glycoside Hydrolase categories from Berlemonte and Martiny 2015.

## Data Availability

The metagenome-assembled genomes (MAGs) generated in this study are available under NCBI BioProject accession PRJNA860067. All data generated or analyzed during this study are included in this published article and its supplementary information files.
