# Supplementary material for: HT-SIP: a semi-automated stable isotope probing pipeline identifies cross-kingdom interactions in the hyphosphere of arbuscular mycorrhizal fungi
Source: Microbiome. 2022 Nov 25;10:199. doi: 10.1186/s40168-022-01391-z (PMC9700909; doi:10.1186/s40168-022-01391-z)
Supplement: Supplementary file 2 — Additional file 1: Supplemental Figure S1. Automated fractionation system design, parts, and assembly. Supplemental Figure S2. Hyphosphere-SIP experimental design. Supplemental Figure S3. Myxococcota MAG CAZymes grouped with CAZymes from 25 genomes from Myxococcota GTDB type species (previously used in Murphy et al. [2]). [file 40168_2022_1391_MOESM1_ESM.docx]

**SUPPLEMENTAL FIGURES**


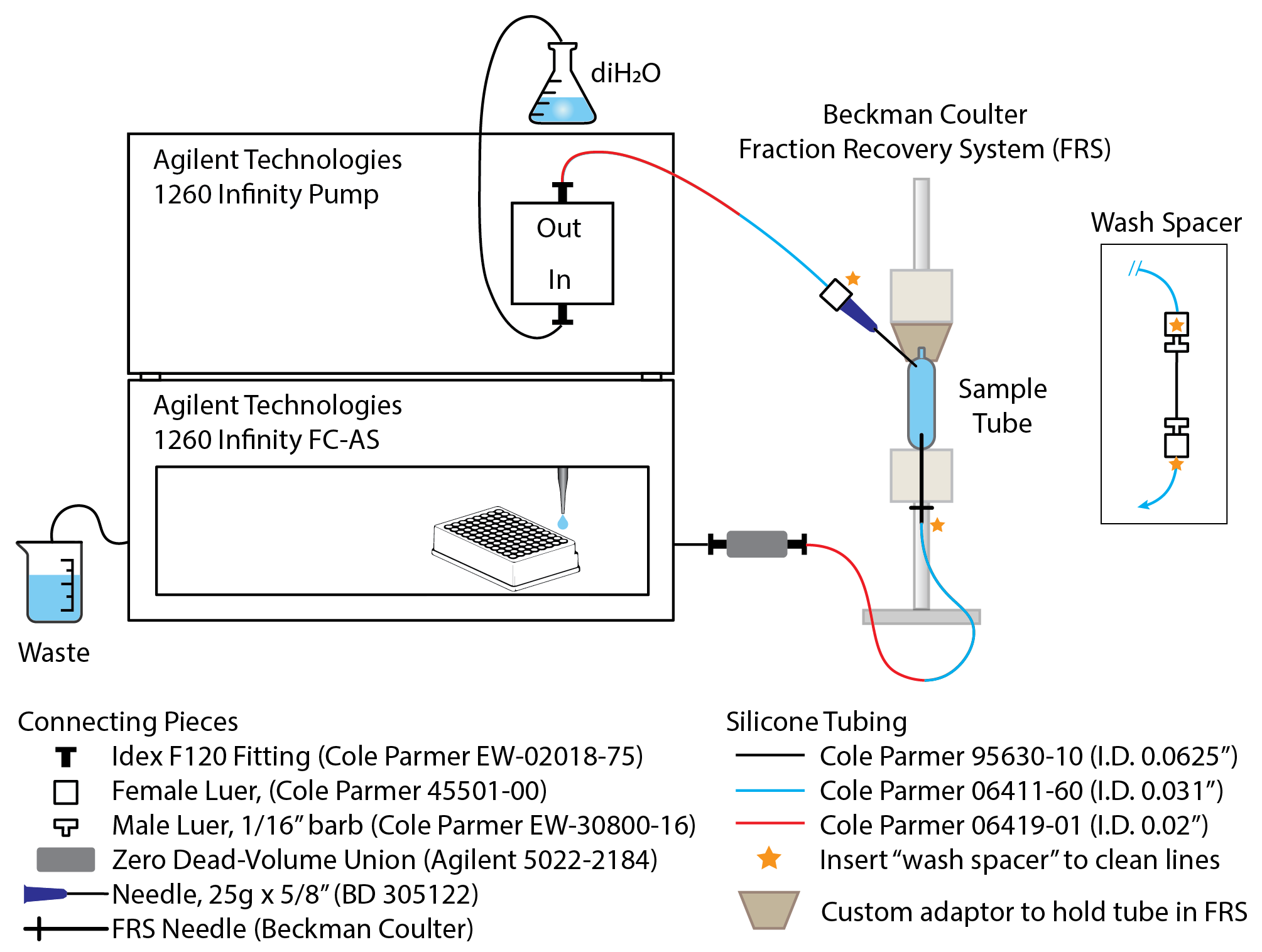


**Supplemental Figure S1. Automated fractionation system design, parts, and assembly.** We automated density fractionation by connecting an Agilent Technologies 1260 Infinity Fraction Collector to a Beckman Coulter Fraction Recovery System (FRS); part numbers are provided for the tubing and connectors required to build this system. The Agilent Infinity pump displaces the gradient buffer with a displacement fluid (e.g., sterile deionized water), and the gradient buffer fractions are collected in a 96-well plate. We attached a custom adaptor to the FRS to hold the gradient tubes in place, since we do not flow the displacement fluid through the FRS directly, but instead puncture the top of the tube with a needle and inject displacement fluid directly into the SIP sample tube. To create the custom adaptor, we use a rubber stopper or cork with a hole on the bottom hollowed out to hold the tube neck, and a piece of plastic (e.g., syringe bore) inserted into the top of the stopper that slots into the FRS gasket; we then hold the tube in place by compressing the FRS upper mount and this adaptor on top of the tube. To clean the tubing after fractionating a sample, the tubing is detached from the needles at the yellow stars and then attached to the wash spacer (attaches to yellow stars on inset diagram); the tubing is then flushed with water and the wash water is collected as waste. Mineral oil can also be used as a displacement fluid.


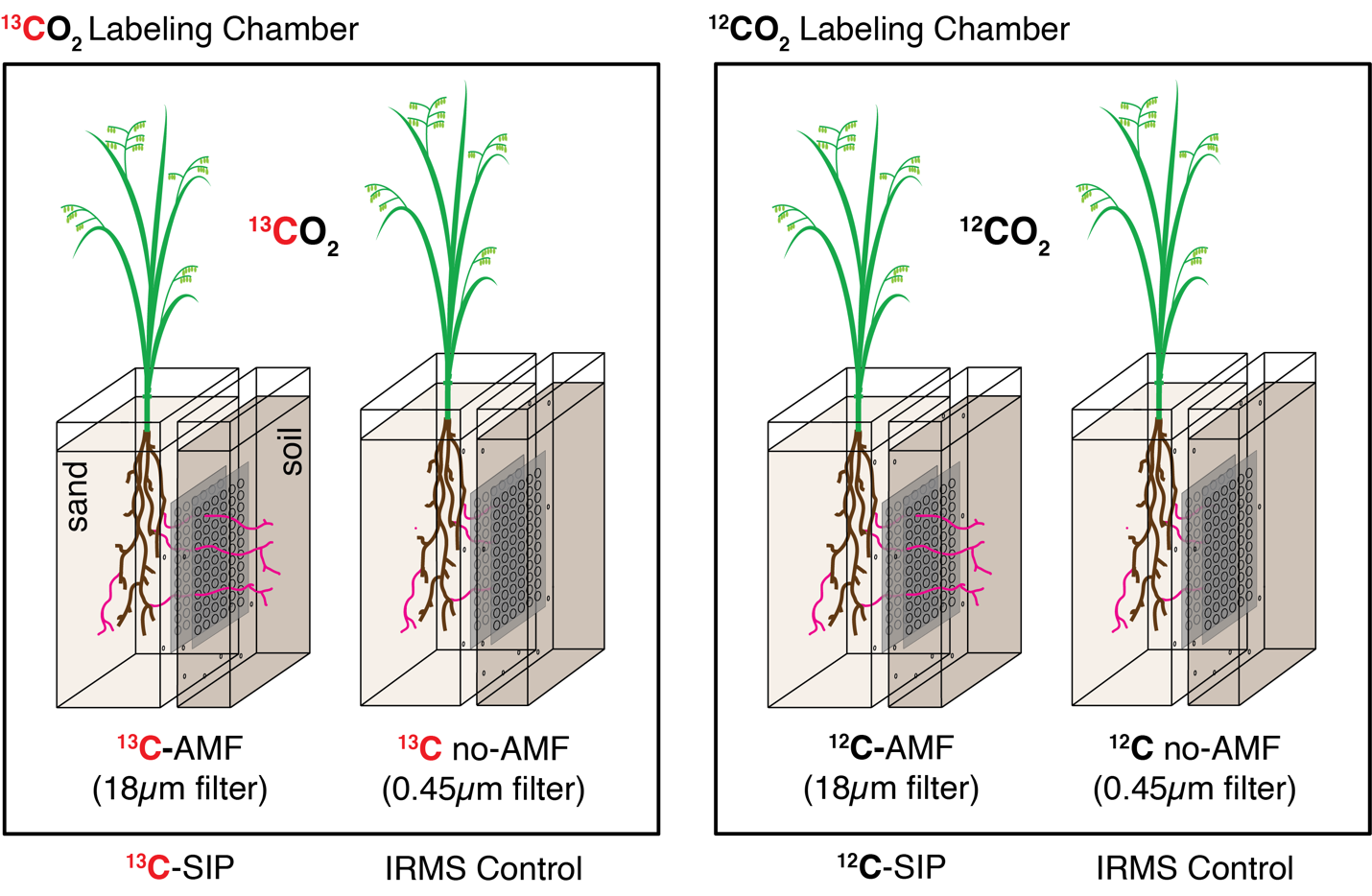


**Supplemental Figure S2. Hyphosphere-SIP experimental design.** A two-compartment microcosm design was used to generate ^13^C-hyphosphere soil for DNA-SIP. In the ‘plant compartment,’ the annual wild grass *Avena barbata* was inoculated with the AMF *Rhizophagus intraradices* in sterile sand. The plant compartment was separated from the ‘no-plant compartment’ by a 3.2 mm air gap, where this second chamber contained live soil only accessible by AMF hyphae. Both sides of the air gap had nylon mesh that either allowed hyphae but excluded roots (‘AMF,’ 18 μm mesh), or that excluded both hyphae and roots (‘no AMF,’ 0.45 μm mesh). Microcosms were placed in isotope labeling chambers with an enriched ^13^CO_2_ or natural abundance ^12^CO_2_ headspace and labeled continuously for 6 weeks. Following labeling, the no-plant compartments were harvested, and hyphae-soil aggregates (AMF permitted, ‘AMF’) and soil (AMF excluded, ‘no-AMF’) were collected. The ^13^C- and ^12^C-AMF samples were used for DNA-SIP analysis (n=3 each), while the ^13^C and ^12^C no-AMF samples were used as IRMS controls (n=3 each) (e.g., to determine if ^13^C entered the soil via a non-mycorrhizal route, such as gas diffusion). Microcosm images were modified with permission from Kakouridis et al. [1].


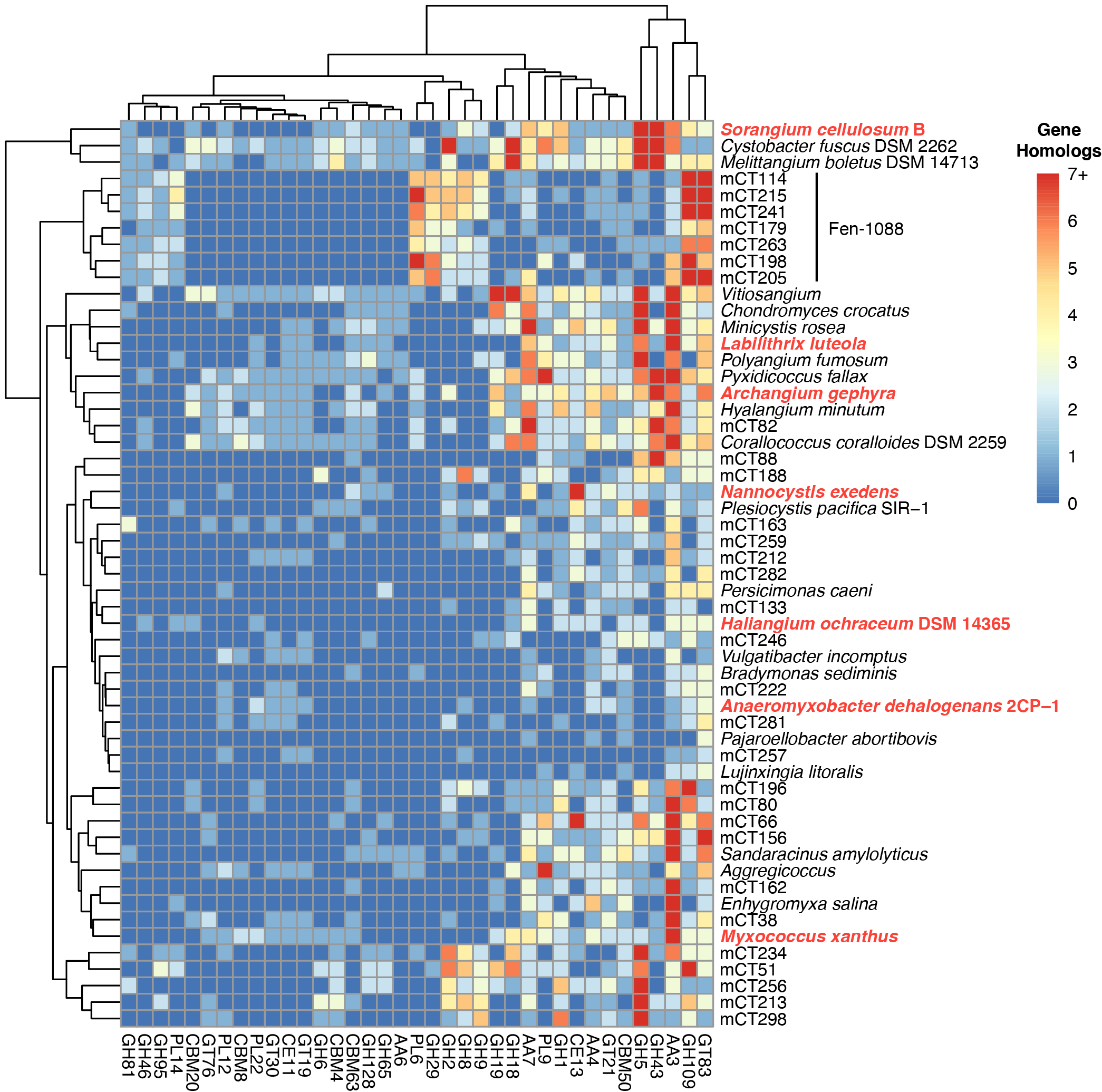


**Supplemental Figure S3**: Myxococcota MAG CAZymes grouped with CAZymes from 25 genomes from Myxococcota GTDB type species (previously used in Murphy et al. [2]). CAZymes and genomes were grouped on the x- and y-axes using one-dimensional hierarchical clustering. Taxa in bold red text were selected as representatives for the hierarchical clusters used in Figure 5. CAZymes displayed have a gene content that was significantly different between the 25 Myxococcota type genomes and Fen-1088 MAGs (two-tailed t-test, p < 0.001). The 25 Myxococcota type species and their NCBI genome assembly accessions are: *Anaeromyxobacter dehalogenans* 2CP-1 (GCF_000022145.1), *Haliangium ochraceum* DSM 14365 (GCF_000024805.1), *Plesiocystis paciﬁca* SIR-1 (GCF_000170895.1), *Corallococcus coralloides* DSM 2259 (GCF_000255295.1), *Cystobacter fuscus* DSM 2262 (GCF_000335475.2), *Hyalangium minutum* (GCF_000737315.1), *Sandaracinus amylolyticus* (GCF_000737325.1), *Archangium gephyra* (GCF_001027285.1), *Chondromyces crocatus* (GCF_001189295.1), *Vulgatibacter incomptus* (GCF_001263175.1), *Labilithrix luteola* (GCF_001263205.1), *Minicystis rosea* (GCA_001931535.1), *Melittangium boletus* DSM 14713 (GCF_002305855.1), *Nannocystis exedens* (GCF_002343915.1), *Bradymonas sediminis* (GCF_003258315.1), *Lujinxingia litoralis* (GCF_003260125.1), *Polyangium fumosum* (GCF_005144585.1), *Persicimonas caeni* (GCF_006517175.1), *Pyxidicoccus fallax* (GCF_012933655.1), *Vitiosangium* (GCF_003044305.1), *Aggregicoccus* (GCF_009659535.1), *Pajaroellobacter abortibovis* (GCF_001931505.1), *Enhygromyxa salina* (GCF_002994615.1), *Sorangium cellulosum* B (GCF_000067165.1), *Myxococcus xanthus* (GCF_900106535.1).

**References**

1. Kakouridis A, Hagen JA, Kan MP, Mambelli S, Feldman LJ, Herman DJ, et al. Routes to Roots: Direct Evidence of Water Transport by Arbuscular Mycorrhizal Fungi to Host Plants. New Phytol. 2022; doi: 10.1111/nph.18281 PMID - 35633108.

2. Murphy CL, Yang R, Decker T, Cavalliere C, Andreev V, Bircher N, et al. Genomes of Novel Myxococcota Reveal Severely Curtailed Machineries for Predation and Cellular Differentiation. Appl Environ Microbiol. 2021;87(23):e01706-21; doi: 10.1128/aem.01706-21 PMID - 34524899.
